# *EGFR* Gene Overexpression Retained in an Invasive Xenograft Model by Solid Orthotopic Transplantation of Human Glioblastoma Multiforme Into Nude Mice

**DOI:** 10.3109/07357907.2010.550665

**Published:** 2011-02-11

**Authors:** Diao Yi, Tian Xin Hua, Huang Yan Lin

**Affiliations:** Neurosurgical Department of Affiliated Zhongshan Hospital, Xiamen University, Xiamen, China

**Keywords:** Glioblastoma multiforme, Orthotopic xenograft, Nude mice

## Abstract

Orthotopic xenograft animal model from human glioblastoma multiforme (GBM) cell lines often do not recapitulate an extremely important aspect of invasive growth and epidermal growth factor receptor (*EGFR*) gene overexpression of human GBM. We developed an orthotopic xenograft model by solid transplantation of human GBM into the brain of nude mouse. The orthotopic xenografts sharing the same histopathological features with their original human GBMs were highly invasive and retained the overexpression of *EGFR* gene. The murine orthotopic GBM models constitute a valuable *in vivo* system for preclinical studies to test novel therapies for human GBM.

## Introduction

Gliomas are the most common forms of primary human brain tumors, and they are often classified into four clinical grades. The most aggressive tumors, grade 4 tumors, also known as glioblastoma multiforme (GBM), are associated with high mortality and morbidity. Survival of patients affected by GBM has remained virtually unchanged during the last decades (i.e., 6–12 months postdiagnosis) despite advances in surgery, radiation, and chemotherapy (1–3). This paradox is notably explained by the impossibility of studying *in vivo* at the cellular and molecular level, the actions of the multiple possible modalities of treatment on human GBM (4). For these reasons, the development of clinically relevant models for studying GBM is essential for increasing our understanding of their tumorigenesis, biology, as well as for testing novel therapeutic approaches for their improved treatment.

Ideal GBM model should recapitulate key features of the human disease, be accurate, be orthotopic, be reproducible, resemble progression kinetics, and retain the important gene alteration, *EGFR* gene overexpression or amplification (5–7). Although rodent glioma models have been used in preclinical glioma research for over 30 years, their use remains controversial and these models have been criticized for not recapitulating main pathological features of human GBM (8). *In vivo* human glioma models developed by subcutaneous (heterotopic) or intracranial (orthotopic) implantation of glioma cell lines in rodents are widely used to test novel therapies for GBM (1, 9–11). The advantages of these glioma models are their highly efficient glioma genesis, reproducible growth rates, and an accurate knowledge of the location of the tumor (3). However, the heterotopic xenografts are not truly representative of the biological characteristics of their original patient GBM, such as invasive growth (4). In addition, in the orthotopic setting, established human GBM cell lines generally also fail to demonstrate the diffusely infiltrative pattern of growth that is typical of human GBM (6); instead, human GBM cell lines tend to form solid masses at the site of injection, which compress rather than invade the surrounding brain parenchyma (12–14). Another major disadvantage of the orthotopic models using xenografted human GBM cell lines in rodents is that genetic alterations present in the original tumor are not often maintained, especially the overexpression or amplification of the *EGFR* gene that is present in approximately 40%–50% of human GBM is typically not preserved in GBM cell lines and xenografts derived thereof (6, 15–17). Consequently, the heterotopic or orthotopic models from human GBM cell lines do not recapitulate an extremely important aspect of tumor invasion and *EGFR* gene overexpression, which has somewhat limited its application in clinically relevant researches.

Alternative methods for establishing orthotopic GBM xenograft models have been more successful at maintaining the invasive features of these tumors, such as the direct transplantation of patient surgical material into the brains of nude mice and transplantation of patient surgical material subcutaneously (sc) in nude mice followed by dissociation and orthotopic reinjection of these xenotransplants (18, 19). Furthermore, the problem of EGFR overexpression loss has previously been overcome by direct implantation of tumor specimens into the flanks of nude mice (19, 20). Consequently, considering the critical value of orthotopic human GBM animal models with high invasiveness and EGFR overexpression in preclinical and translational cancer research, in the present work, we establish the intracranial xenograft models by orthotopic retransplantation of human GBM solid tissues maintained as xenografts via serial passaging sc in the flanks of nude mice and report whether the intracranial xenograft models can retain histopathological features and genetic properties of the clinical GBM with high invasiveness and EGFR overexpression. The preservation of tumor EGFR overexpression status as well as tumor invasiveness in the orthotopic setting will give the opportunity to assess the efficacy of developing novel therapeutic approaches for human GBM.

## Materials and Methods

### Clinical information

**Figure 1:**
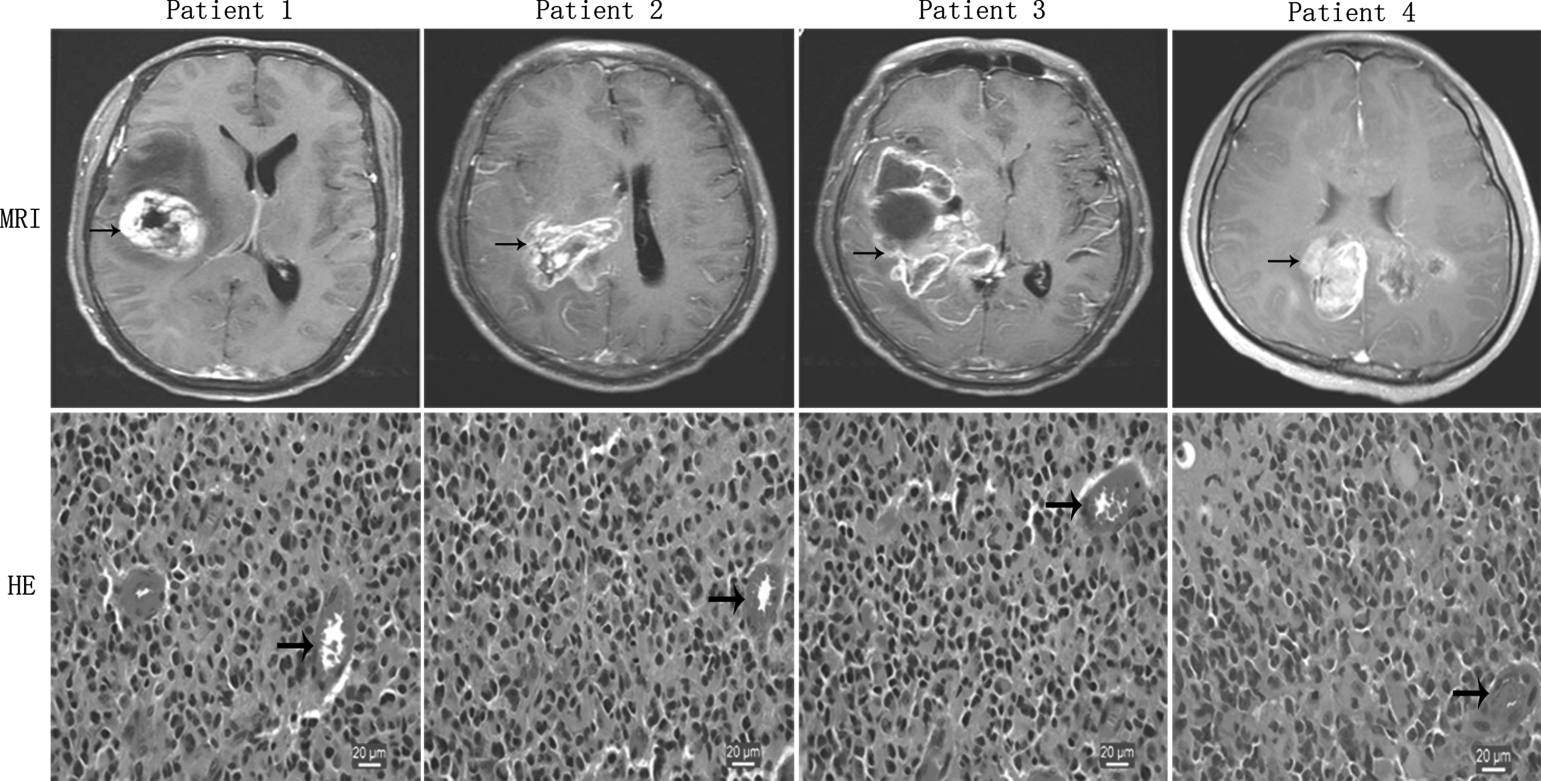
MRI and histopathologic features of human GBMs. MRI reveals irregularly and nonhomogeneously enhancing mass (*black arrow*) in the right hemisphere zone, and edema zone surrounding solid tumor sometime could be detected in contrast-enhanced T1-weighted imaging. Histopathologically, patient tumor morphology is mitotically active and includes pleomorphic cells, nuclear atypia, abundant microvasculars (*black arrow),* endothelial proliferation, and necrotic foci (HE staining).

**Figure 2:**
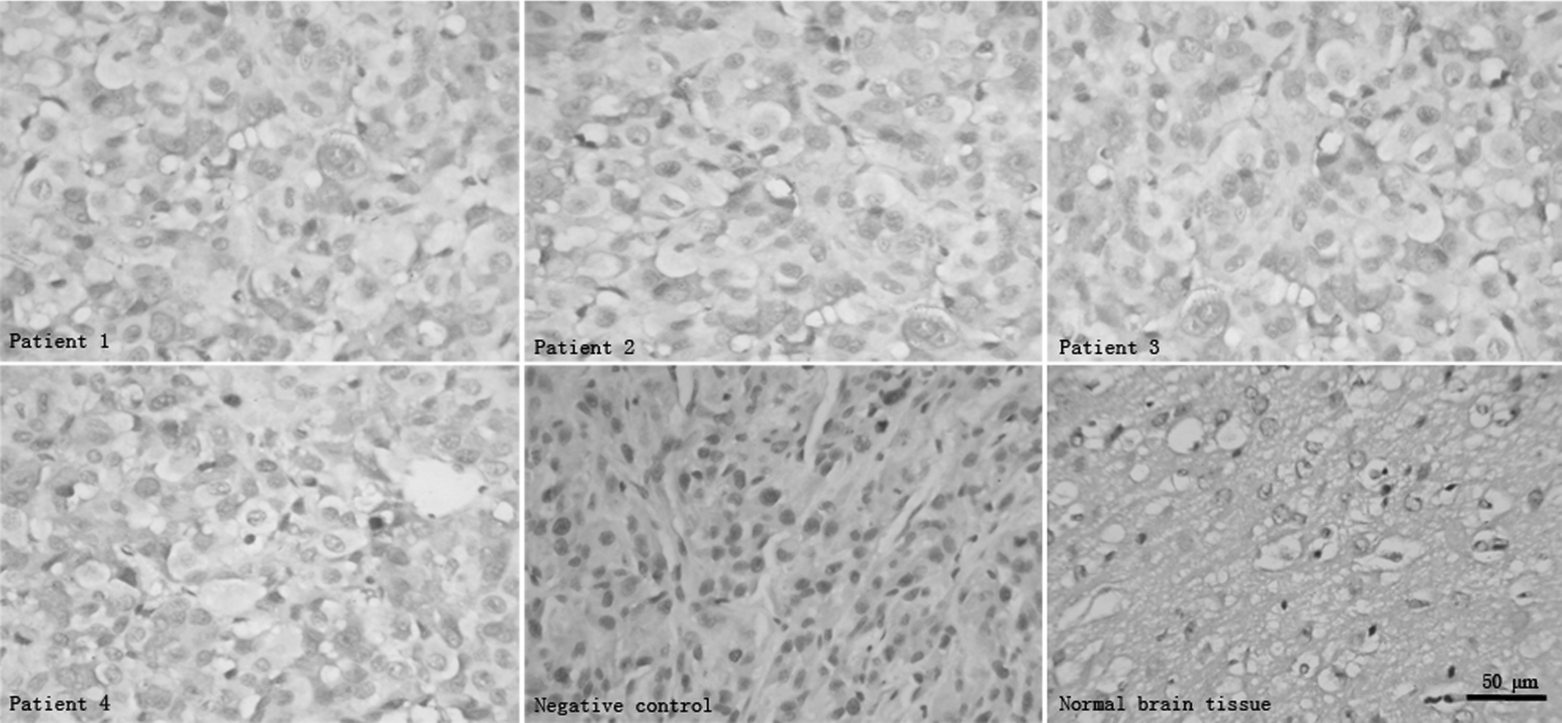
Immunohistochemical analysis of EGFR protein expression in human GBMs. Compared with human normal brain tissues from surgical decompression without EGFR expression, the 4 human GBMs maintain the genetic property of EGFR overexpression. PBS instead of primary antibodies is used as negative controls.

Tumors used in this study were obtained from 4 patients who were undergoing surgical treatment at the Neurosurgical Department of Affiliated Zhongshan Hospital of Xiamen University and who had consented to the use of their tissues for research. Meanwhile, the 4 patients were randomly numbered as 1, 2, 3, and 4. Tumor images in the 4 patients were obtained by using a standard T1 protocol following gadolinium injection under 1.5-tesla clinical magnetic resonance scanner; intraoperative surgical pathology consultation confirmed the suspected clinical diagnosis of GBM with pleomorphic cells, presence of mitotic activity, abundant microvasculars, endothelial proliferation, and necrotic foci (Figure 1). In addition, *EGFR* gene overexpression was also demonstrated in the tumor tissues of the 4 patients by immunohistochemical analysis, of which positive rate of EGFR in the 4 human tumors were 67.5% ± 3.2% (Figure 2).

### Nude mice

Four-to-six weeks old, congenitally athymic nude mice, female, on Balb/c nu/nu background, were purchased and bred in the laboratory animal center of the Xiamen University. Mice were maintained under specific pathogen-free barrier environment, fed with commercial rodent diet, and provided with clean water *ad libitum*. All experimentations and animal usage were performed and approved by the National Science and Technology Committee guidelines (China). For grafting and imaging, the mice were anesthetized intraperitoneally with a 0.10 mg ketamine hydrochloride solution per gram body weight.

### Primary flank implantation

Above excess fresh tissues of 4-patient GBMs were kept in a sterile isotonic glucose solution for initial heterotopic implantation. Namely, the tumor tissues of patients 1, 2, 3, and 4 were used for establishing correspondingly subcutaneous (sc) xenograft lines 1, 2, 3, and 4, respectively (Table 1). The preserved 4-patient tumor tissues were immediately minced into small pieces, placed into an 18-gauge trochar, and injected into sc flanks of mice, respectively. When the sc tumors in the 4 different xenograft lines developed until they reached a length of 1.0–1.5 cm in longest dimension, the mice were sacrificed, and then their flank tumors were immediately excised and retransplanted into sc flanks of another mice. Thus, the original tumors of 4 patients were passaged from animals to animals for 2 years, amounting to 4 generations with 8 mice per generation, 2 mice per sc xenograft line. In addition, part of excised flank tumor tissues of each generation of mice was detected by using HE staining or relevant molecular analysis for EGFR expression.

**Table 1:** Subcutaneous and Intracranial Implantation: Xenograft Origin, Number of Mice and Testing Strategy

Patient tumor number	Subcutaneous xenograft line^a^	Intracranial xenograft line^b^	MRI^c^	Histopathological/molecular tests^d^
1	1	1	10	20
2	2	2	10	20
3	3	3	10	20
4	4	4	10	20

a Subcutaneous xenograft line 1, 2, 3, and 4 derived from corresponding patient tumor 1, 2,3, and 4, respectively, and each patient tumor was xenotransplanted into 2 mice flanks with one graft per mouse.

b Intracranial xenograft line 1, 2, 3, and 4 derived from correspondingly subcutaneous xenograft line 1, 2, 3, and 4, respectively, and grafts in each xenograft line were transplanted into 30 mice brain.

c 10 mice per intracranial xenograft line were scanned for brain MRI.

d 20 mice per intracranial xenograft line were performed for histopathological and relevant molecular analysis.

### Secondary intracranial implantation

Now, the 4th generation of sc GBMs in xenograft lines 1,2,3, and 4 were excised, respectively, and prepared for further orthotopic implantation in order by inoculation into the brains of another nude mice to establish correspondingly intracranial xenograft lines 1,2, 3, and 4 and to observe its biological features (Table 1). Mice were secured to the stereotaxic holder (Huaibei Zhenghua Biologic Apparatus Facilities Co., Ltd., Huaibei, China). The head skin of the surgical site was shaved and disinfected. A 5-mm midline scalp incision was made, and the bregma set was exposed. A small burr hole (1 mm diameter) was made in right frontal bone (1.0 mm anterior and 2.5 mm lateral to the bregma) with microdrill bit. The fragments of sc fresh transplantable tumor specimens were placed into 24-gauge trochar (outer diameter close to 1 mm). Then, the volume of tumor fragments placed into trochar was flexibly adjusted up to 2.0 mm^3^ by the trochar's inner needle. Afterward, the trochar loaded with tumor fragments was vertically and slowly inserted up to a depth of 4.0 mm below the outer table of the skull through the small burr hole just formed in the skull bone. After pulling the trochar back 1.0 mm, tumor fragments were slowly pushed out by trochar's inner needle to make sure that tumor fragments were completely implanted into the right caudate nucleus. The skull hole was tamped with bone wax, and skin was stitched. Note that 120 mice were used for orthotopic transplantation with 30 mice per xenograft line. Furthermore, the flank-derived nontumor tissues were also orthotopicly placed into brains of another 4 groups of mice with 10 per group as sham surgical controls, namely, negative controls. Following tumor tissues injection, the subjective mice were treated subcutaneously with 2.5 mg/kg flunixin meglumine (Qilu Pharmaceutical Co., Ltd., Jinan, China) with one time per day for 3 days to alleviating the postoperative suffering of animals. Mice were observed daily until they reached a cachectic state in the surgical groups; then all mice including surgical and sham surgical groups were scanned by magnetic resonance scanner for brains, or sacrificed, and their brains were removed and processed for histopathologic analysis and relative molecular detection of EGFR expression.

### Magnetic resonance imaging

Ten mice per intracranial xenograft line (Table 1) were scanned for brain imaging when they became cachectic by Philips Achieva 1.5 T clinical magnetic resonance imaging (MRI) appliance with microcoil (inner diameter, 2.3 cm). Mice scanned were anesthetized with a ketamine hydrochloride solution (0.10 mg/g) by ip injection. The contrast material, gadopentetic acid dimeglumine (Magnevist, Guangzhou, China) 0.5 mL, was injected ip 10 min before examination. The console settings chosen to optimize signal-to-noise ratio and spatial resolution were as follows: image matrix, 224 × 224 and slice thickness, 3.0 mm. A T1 spin echo pulse sequence was used with a repetition time of 260 ms. The echo time was 24 ms with two excitations. The duration of this sequence was 90 s. Axial, coronal, and sagittal 3.0-mm slices were obtained per brain with 0.3 mm of interslide. In addition, magnetic resonance scanning was also performed for the mice brains as sham surgical controls.

### Histopathological analysis

Twenty mice per intracranial xenograft line (Table 1) were sacrificed when they became cachectic. Whole brains of mice were removed from the cranial cavity, bisected coronally at the innoculation site. Half of the bisected brains were fixed by overnight immersion in formalin, and the fixed specimens were subsequently embedded in paraffin and then sectioned (6-µm-thick) according to routine pathological procedures for morphological studies; the remainder of the bisected brains was frozen in isopentane precooled in liquid nitrogen for further relative analysis. Mice brains as sham surgical controls were also performed for the same pathological procedures. HE staining procedures are summarized below: Sectioned tissues were deparaffinized and then hydrated in distilled water. Hydrated sections were immersed in hematoxylin, then counterstained with eosin, and finally cleared with two changes of xylol prior to mounting.

### Immunohistochemical analysis

Above paraffin-embedded sections were immunostained for detecting the presence of total EGFR protein and the development of tumor vasculature with corresponding monoclonal antibody against human EGFR or mouse CD34. Staining for EGFR protein was accomplished with microwave antigen retrieval in 10 mM sodium citrate, pH 6.0, followed by the cooling of tissue sections to room temperature prior to adding rabbit monoclonal antibody against EGFR protein (1:200 dilution; DAKO, Glostrup, Denmark), with subsequent overnight incubation at 4°C. Furthermore, each generation of flank xenografts was also performed for the procedure. Mice brains and flank-derived nontumor tissues in the sham surgical groups were as normal control, and PBS instead of primary antibodies was used as negative controls. Staining of cell membrane for EGFR was scored as follows: negatively stained cell is less than 25%, weakly positive is 25%-50%, positive is 50%–75%, and strongly positive is more than 75% stained cells (21, 22). To detect tumor angiogenesis, sections were stained with a rabbit monoclonal antibody (1:100 dilution; DAKO, Glostrup, Denmark) against mouse CD34 antigen for labeling endothelial cells of microvessels (23). All above staining was visualized by use of the Dako En Vision kit according to the manufacturer's instructions.

### Western blot analysis

To investigate the expression of EGFR protein in intracranial GBM xenografts and its original tumors, we employed a western blot method. Above portion of frozen intracranial, subcutaneous xenografts and patients' tumor tissues were lysed with ice-cold 20 mM Tris-HCl containing protease inhibitors. Lysates (100µg of protein) were separated on a 10% polyacrylamide gel under reducing conditions and blotted onto an Immobilon-P membrane. Blots were blocked with 5% nonfat dry milk in PBS containing 0.2% Tween 20 and incubated with rabbit monoclonal antibody against human EGFR (1:200) overnight at 4°C. After washing, filters were incubated with a goat antirabbit antibody conjugated with horseradish peroxidase at room temperature for 20 min. The blot was developed with enhanced chemiluminescence reagents. Brain tissues from human specimens of surgical decompression without EGFR expression were as normal control.

### Reverse transcriptase-polymerase chain reaction analysis

To investigate the EGFR expression level in the intracranial GBM xenografts and its original tumors, we employed a reverse transcriptase-polymerase chain reaction (RT-PCR) assay using EGFR primers. Total RNA was extracted from the frozen intracranial, subcutaneous xenografts and patients' tumor tissues. Integrity of the RNA is demonstrated by a high-resolution gel method. After the reverse transcription, EGFR and β-actin primers were used for cDNA amplification. PCR products were electrophoresed on agarose gels containing ethidium bromide and visualized by UV photography. Primer sequences for human EGFR and β-actin were as follows: sense, 5′-AAGGCTGTCCAACGAATGGG-3′ and antisense, 5′-CCTCTCCTGCAGCAGCCTC-3′ for EGFR resulting in a 150-bp PCR product; sense, 5′-CACCAACTGGGACGACATG-3′ and antisense, 5′-GCACAGCCTGGATAGCAAC-3′ for β-actin resulting in a 250-bp PCR product (22). No template for RT-PCR was as negative control. Brain tissues from human specimens of surgical decompression without EGFR expression were as normal control.

### Methods of Data Analysis

Difference in the numbers for necrotic foci, vascularization, invasion and percentages for EGFR protein expression between 4 different intracranial xenograft lines was analyzed using one-way ANOVA.

## Results

### Histopathology of flank xenografts

**Figure 3:**
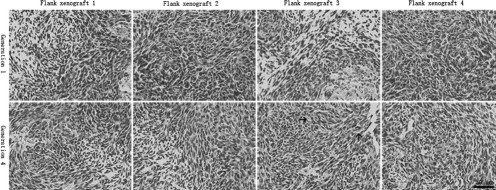
Histopathologic features of flank xenografts. Compared with the human surgical materials, GBM xenografts in 4 different xenograft lines displayed pleomorphic cells, presence of mitotic activity, necrotic foci, and mild microvasculars (black arrow), but endothelial proliferation with multilayering of endothelial cells was not observed.

All flank GBMs of 4 generations in 4 different xenograft lines displayed pleomorphic cells, presence of mitotic activity, necrotic foci, and mild microvasculars (Figure 3). Of 32 flank tumors, 26 showed evidence of necrosis, and the extent of necrosis appeared to be proportional with increasing flank tumor size. Mild microvascular proliferation was observed in only five instances. Endothelial proliferation with multi-layering of endothelial cells was not observed. Furthermore, immunoreactivity for EGFR protein was noted in all flank xenografts. Of 32 flank xenografts, positive rate of EGFR in 24 cases was 59.5% ± 4.5% and in 8 cases was 78.6% ± 5.2%. Compared with the human surgical materials (Figures 1 and 2), the serial mouse-grown flank tumors retained the same morphological cell types and biological features of EGFR overexpression.

### Intracranial xenografts take

All the mice with intracranial xenotransplantation survived the surgical procedures, and the animals' clinical status was in all cases normal after the recovery from anesthesia. All mice grafted intracranially from 4 different flank xenograft lines appeared cachectic until the end of the observation period, while those grafted intracranially from flank-derived nontumor tissues did not appear cachectic. All cachectic mice were confirmed to have developed tumors by the naked eye, successive HE staining or MRI, while no grafts appeared in the brains of mice without cachexia in the sham surgical controls. Thus, each intracranial xenotransplantation from 4 different flank xenograft lines produced a tumor with 100% taking rate. Although mice received orthotopic xenotransplantation from 4 different flank xenograft lines, respectively, the survival time of graft-bearing mice in the 4 different intracranial xenograft lines was consistent, remaining 20.6 ± 1.8 days, which suggests that our established orthotopic GBM model is stable and provides a foundation for experimental treatment of GBM.

### Neuroimaging

**Figure 4:**
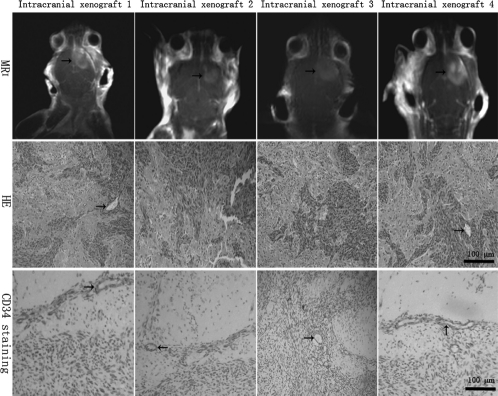
MRI and histopathologic features of intracranial xenografts. The 4 different intracranial xenografts generated from 4 corresponding flank GBM xenograft lines. MRI reveals irregularly and nonhomogeneously enhancing mass (*black arrow*) in contrast-enhanced T1-weighted imaging, which shared the similar MRI features with its corresponding human tumors. In HE-stained sections, the intracranial xenografts show mitotically active, high cellular density, poorly differentiated pleomorphic cells, necrotic foci, and some multinucleated cells, as well as mild or profuse microvessels (*black arrow*). Microvessels appear to consist of a continuous single layer of endothelial cells in CD34 immunostaining (*black arrow),* but endothelial proliferation is not observed compared with human tumors.

On each occasion, the postgadolinium T1-weighted sequences of the mice brain revealed typical intense contrast enhancement in the right hemisphere of 10 mice per intracranial xenograft line. The contrast-enhanced zones corresponded to the solid tumor component, and edema zone surrounding solid tumor sometime could be detected (Figure 4). The intracranial xenografts shared the similar MRI features with its corresponding human tumors (Figure 1). However, no enhancing masses were observed in the brains of mice in the sham surgical controls (data not shown).

### Histopathology of intracranial xenografts

In HE-stained sections, features common to the intracranial GBMs in 4 different xenograft lines included high cellular density with frequent nuclear crowding, poorly differentiated pleomorphic cells, some multinucleated cells, and frequent mitoses. Compared with the human surgical material (Figure 1), the mouse-grown intracranial tumors contained the same morphological cell types, but there was a tendency for the tumor cell composition to appear somewhat more homogeneous (Figure 4).

No significant difference in the numbers of necrotic foci was observed between the 4 xenograft lines (Table 2), and the necrotic foci were often surrounded by pseudo-palisading of tumor cells. The presence of microvascular/endothelial proliferation (i.e., multilayered, mitotically active hyperplasic endothelial cells, smooth muscle cells, and pericytes) is a histopathological hallmark of human GBM (24, 25), but endothelial proliferation with multilayering of endothelial cells was not observed in any intracranial xenografts. In sections stained with the monoclonal antibody to mouse CD34 antigen, the microvessels of intracranial xenografts appeared to consist of a continuous single layer of endothelial cells (Figure 4). No significant difference in the numbers of vascular-ization was observed between the 4 xenograft lines (Table 2). Glomeruloid body-like vasculature formation that occurred in corresponding human GBM was never observed. Vascularization observed in the intracranial xenografts was phe-notypically distinct from the human GBM.

**Table 2:** Difference in Numbers for Necrotic Foci, Vascularization, Invasion and Percentages for EGFR Protein Expression Between 4 Different Intracranial Xenograft Lines

	Xenograft Line 1	Xenograft Line 2	Xenograft Line 3	Xenograft Line 4	*p* value
Necrotic foci	1.5 ± 1.6	1.2 ± 1.6	1.0 ± 1.3	1.4 ± 1.5	*p* >.05
Vascularization^a^	6.2 ± 1.0	5.7 ± 0.8	6.1 ± 0.9	5.9 ± 0.9	
Invasion^b^	10.0 ± 1.4	9.7 ± 1.7	9.0 ± 1.8	9.8 ± 1.6	
EGFR	69.8 ± 2.9	68.4 ± 2.6	68.5 ± 3.5	67.4 ± 1.2	

a Vascularization was determined by counting the microvessel density in the intracranial xenografts.

b Invasion was determined by counting the number of small satellite tumors surrounding the intracranial xenografts.

**Figure 5:**
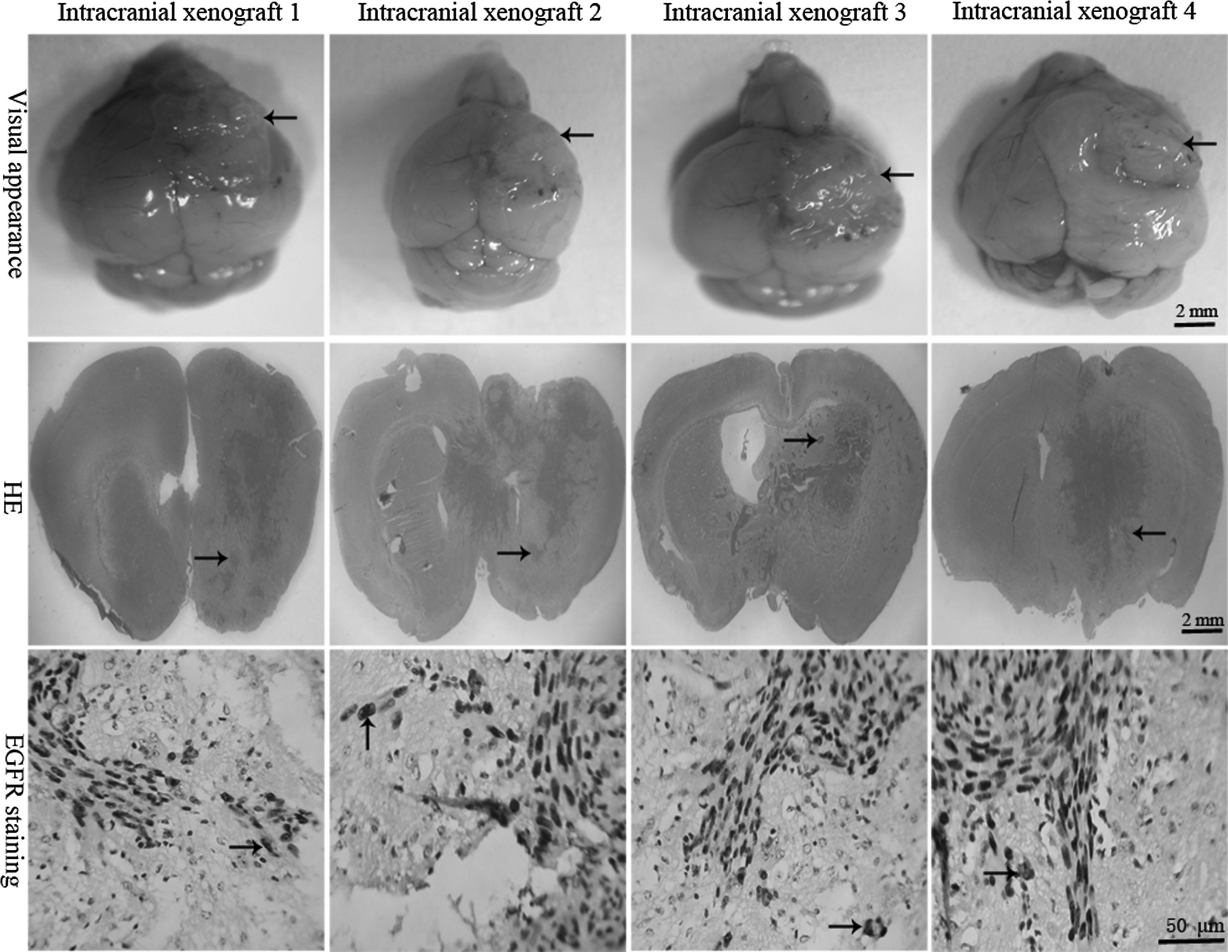
Highly invasive characteristics of intracranial xenografts. The 4 different intracranial xenografts generated from 4 corresponding flank GBM xenograft lines. Macroscopically, tumors (*black arrow*) grow up to the surface of ipsilateral cortex, blurring the border with surrounding normal host brain. In HE-stained sections, a large number of tumor cells can be seen migrating through the corpus callosum and extending into the opposite hemisphere, and the migrating tumor cells (*black arrow*) are clearly entering the normal host brain tissue, suggesting an invasive phenotype of intracranial GBM xenografts. EGFR immunostaining shows that single or clusters of tumor cells (buffy, *black arrow*) are infiltrating the surrounding normal host brain parenchyma.

High invasive growth pattern was a striking feature of the intracerebral xenografts (Figure 5). All xenografts appeared to be composed of two components: solid tumor tissue and invasive tumor cells. The invasive tumor cells, isolated or grouped in nests, could be observed infiltrating surrounding parenchyma from the inoculation site with distant extension away from the solid tumor mass. Reflecting their invasive behavior in the patient setting, the invasive tumor cells of intracranial xenografts were observed as migrating along white matter fibers of the corpus callosum, as well as through the anterior commissure, leading to tumor dissemination to the contralateral hemisphere. Infiltration of adjacent cortex and basal ganglia was also evident. However, intraventricular tumor spread was not observed. No significant difference in the numbers of small satellite tumors indicating the invasion was observed between the 4 xenograft lines (Table 2). Ultimately, the nearly entire cerebral hemisphere was infiltrated by the invasive tumor cells. Because the transplant involved the placement of a small tumor fragment into the mouse brain, no intratissular artificial diffusion of the malignant cells can appear, as can occur during intracerebral injections of cell suspensions (26, 27).

The migration of tumor cells in the normal host cerebral tissue is easy to identify by using immunostaining techniques or HE staining. Pleomorphic cells with abnormal cytoplasmic and nuclear morphology infiltrating the nonneoplastic brain parenchyma showed EGFR-positive expression (buffy), and they had strongly acidophilic cytoplasm, high nucleus to cytoplasm ratio, and nuclear and cytoplasmic abnormal morphology, indicating that the pleomorphic cells were tumor cells (Figure 5). The extent of normal brain invasion by the neoplastic astrocytes varied from tumor to tumor, but the characteristics of the migration of malignant cells in human and murine brains are similar. It corresponds not only to the capacity of malignant glial cells to spread but also to the organization of the cerebral tissue, since the same cells do not spread when they are in contact with other mouse tissues as is the case with our sc heterotopic transplantations. The model of intracranial transplantation into nude mice reproduces the behavior of malignant cells in the human brain. However, no grafts appeared in the brains of mice in the sham surgical controls.

### Immunohistochemical analysis of EGFR expression in intracranial xenografts

**Figure 6:**
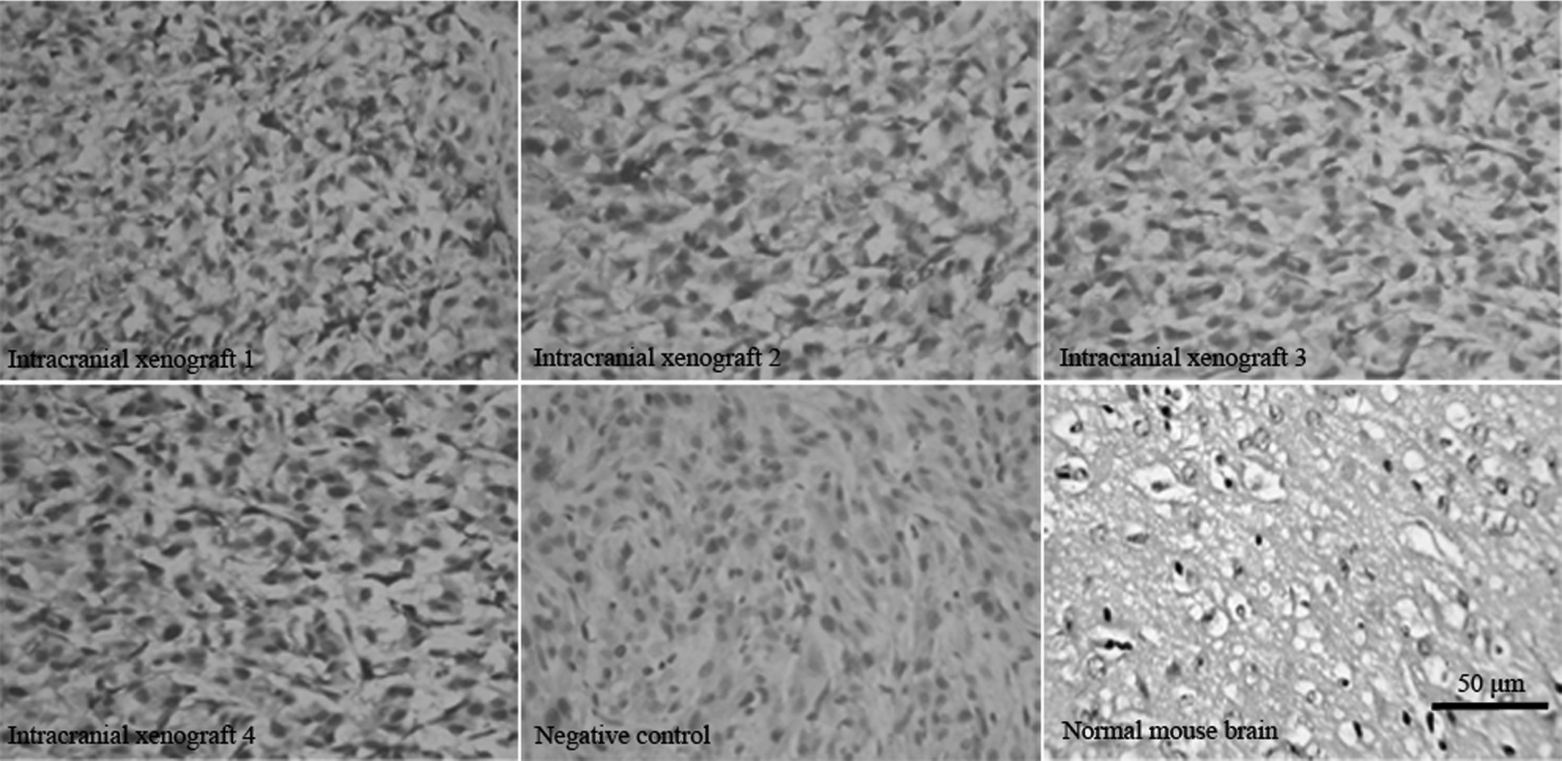
Immunohistochemical analysis of EGFR protein expression in intracranial xenografts. Compared with normal mice brains tissues without EGFR expression, the 4 different intracranial xenografts generated from 4 corresponding flank GBM xenograft lines contain overexpressed EGFR protein. Compared with Figure 2, the intracranial xenografts retain the genetic property of human EGFR overexpression. PBS instead of primary antibodies is used as negative controls.

Immunoreactivity for EGFR protein was noted in all intracranial xenografts. Positive rates of EGFR expression were 69.8% ± 2.9%, 68.4% ± 2.6%, 68.5% ± 3.5%, and 67.4% ± 1.2% in the 4 different intracranial xenografts, respectively, and of which no significant difference in the precentages of EGFR protein expression was observed between each line (Table 2). However, EGFR overexpression features of human GBMs did not appear in the mice brains from sham surgical controls (Figure 6). Compared with the human surgical materials (Figure 2), the mouse-grown intracranial tumors retained the biological features of EGFR overexpression.

### EGFR transcription and expression

**Figure 7:**
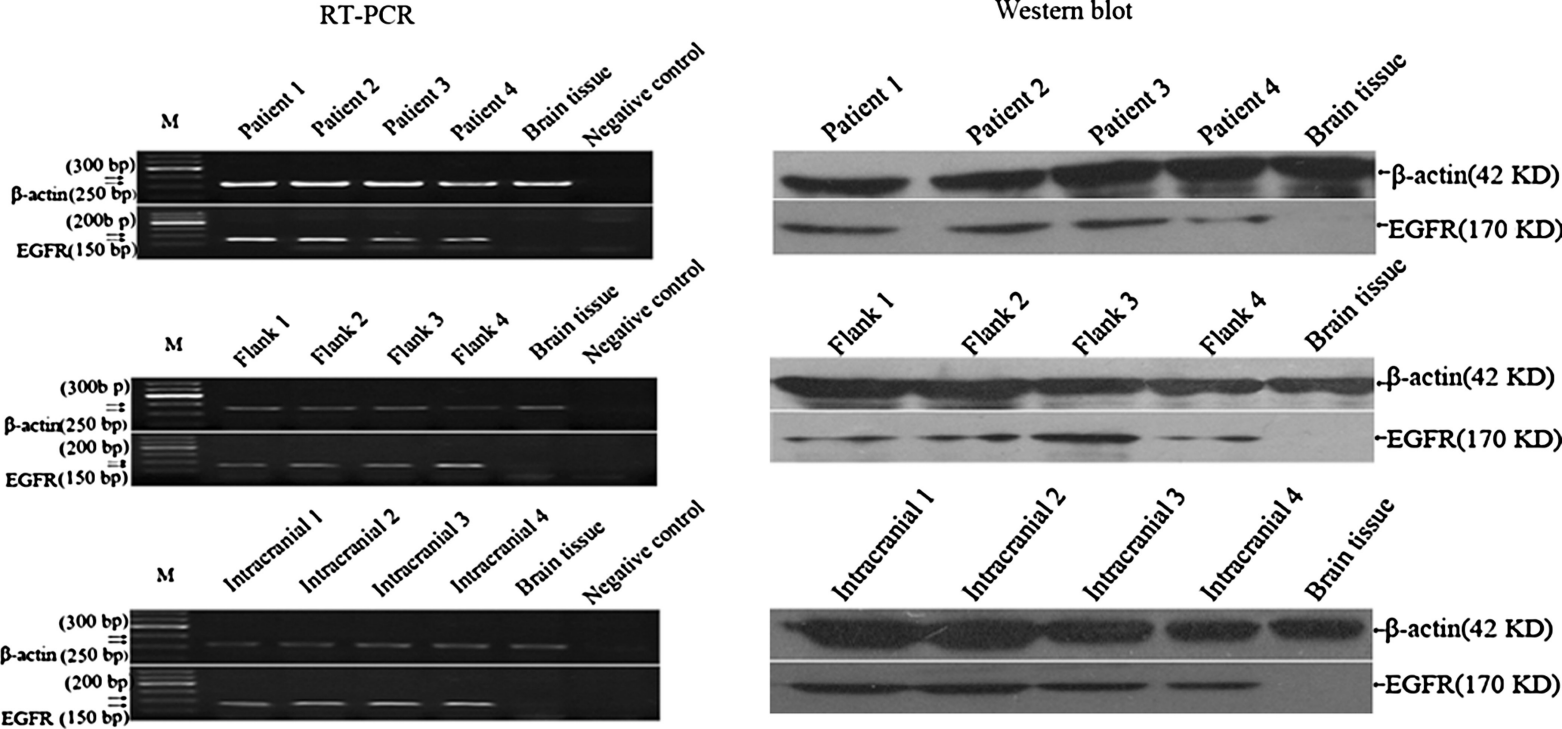
EGFR transcription and expression in xenografts and its original tumors. Compared with human tumors, the overexpressed *EGFR* gene is retained in the GBM xenografts of 4 different lines by RT-PCR and western blot analysis. No template for RT-PCR is as negative control. Brain tissues from human specimens of surgical decompression without EGFR expression are as normal control.

High EGFR transcription level was found in the original patient's GBM tumor and was maintained in all subsequent xenograft lines (flank and cranial) by RT-PCR, and overexpressed EGFR protein was also found in all aforementioned GBM xenografts and its human GBMs by immunoblotting (Figure 7). Here, our RT-PCR and western-blot analysis demonstrated the presence of *EGFR* gene overexpression in all GBM xenografts compared with its original tumors, suggesting that the intracranial or flank xenograft models can retain the genetic property of EGFR overexpression in clinical GBM by orthotopic or heterotopic retransplantation of human GBM solid tissues.

## Discussion

Human GBM is one of the most devastating cancers. Extensive tumor cell invasion occurs in normal brain parenchyma, making it virtually impossible to remove the tumor completely by surgery and inevitably causing recurrent disease. This characteristic of high invasiveness certainly contributes to the failure of current therapies aimed at trying to control this aggressive malignancy. Therefore, there is a compelling need for more reliable *in vivo* preclinical models for studying the disease and for testing new drugs and therapies, and this reliable model should replicate human GBM biological properties with high invasiveness and overexpression of *EGFR,* one of major genes relevant to its high malignancy. However, the limited number of preclinical models that recapitulate the invasive GBM tumor growth with the overexpression of EGFR is a major hurdle to develop new therapies for GBM. Most established GBM cell lines form discrete, non-invasive tumors with well-circumscribed borders that push aside rather than invade adjacent normal tissue (12–14, 28) and cannot maintain the overexpression of the key *EGFR* gene that can promote glioma cell invasion (15–17, 29, 30). This lack of invasiveness and EGFR overexpression may limit the clinical relevance of studies assessing the efficacy of novel therapies aimed at EGFR and invasion when tested against the intracranial xenografts established from GBM cell lines.

Intracranial GBM xenografts established directly from patient surgical specimens that were maintained as sc xenografts through serial passaging in immune-deficient mice have been previously described (20). This approach to establish GBM xenograft is the only means that has been shown to preserve tumor EGFR overexpression status and invasive growth pattern when compared with the established cell lines (18, 20, 31). In our study, all of the intracranial xenografts that we have tested formed highly invasive tumors that show widespread dissemination, infiltration along white matter tracts to the contralateral hemisphere, and even extension along the leptomeninges. The invasive properties of human GBM xenotransplanted orthotopically very closely resemble those of GBM in humans. Because the GBM xenografts that have been continuously propagated as flank tumors recapitulate this very important and characteristic feature of human GBM with high invasiveness following intracranial transfer, the heterotopic-to-orthotopic tumor propagation model should provide a more relevant system for preclinical assessment of novel therapeutic agents, especially for those agents targeting the invasive phenotype of GBM (32).

The genetic characterization of all intracranial GBM xenografts is another key aspect of our model, which retained the overexpression of *EGFR* gene. EGFR is a member of the tyrosine kinase family of cell surface receptors and demonstrates various levels of expression throughout the cellular development and in a variety of different cell types. EGFR has been implicated in human cancers, where it may contribute to both the initiation and progression of the disease. It is frequently present in an amplified or overexpressed form in up to 30%–40% of malignant gliomas, involving glioma cell proliferation and invasion (6, 33). Here, we have shown that the overexpressed EGFR identified in intracranial xenografts is consistent with that determined in corresponding patient GBM. Because the EGFR alteration was also stably retained in sc serial passaging of GBM xenografts, it is clear that flank tumor gene alteration was maintained following tumor transfer to the orthotopic setting. Thus, immunodeficient rodents used for the heterotopic propagation and serial passaging of GBM can be considered as reservoirs for providing a continuing supply of the same tumor for establishing orthotopic model that retains the *EGFR* gene alteration when compared with previous reported methods (34, 35).

Our intracranial GBM xenografts exhibited histological features that are similar to those of the human GBM, including the presence of angiogenesis, necrosis, and the astrocytic phenotype, which showed that the histopathological features of human GBM can be really replicated by heterotopic-to-orthotopic tumor tissues xenotransplantation, although vas-cularization was phenotypically distinct from the human GBM. Vascularization is a characteristic of GBM and associated with poor prognosis (36). Profuse or mild tumor neovas-cularization was observed in our intracranial xenografts, but it is important to emphasize that these intracranial xenografts generally do not display any endothelial proliferation. The absence of endothelial proliferation could be a result of the lack of extracellular matrix components involved in endothelial cell proliferation and migration in the murine host (37, 38). Nevertheless, these models were widely applied and proved useful when assessing GBM angiogenesis and antian-giogenic therapeutic approaches (6, 39), as shown above (data not shown). Necrosis is a hallmark of glioblastoma occurring in 60% of GBM patients (40). In our study, necrosis was observed in 35 cerebral xenografts whose largest diameter was in excess of 5.5 mm, but observed in most of our passaging flank tumors. Because the flank tumors can be grown to substantially larger volumes than their corresponding intracranial tumors, our results suggest that the development of necrosis could well be dependent on the tumor having achieved sufficient size. This hypothesis could be tested via tumor propagation in an animal with a larger intracranial volume, such as a dog (8).

Real-time noninvasive imaging technologies permit longitudinal monitoring of tumor progression. MRI is commonly used for human brain tumor imaging, but it has been refined in preclinical models. The use of MRI to study the spontaneous evolution of gliomas implanted in the brains of dogs, rats, or mice has been performed for many years (4, 41). Our findings indicate that this technique is sensitive enough to allow evaluation of tumor growth in the mice brains. In spite of the small size of the mouse brain and of the tumors used here, there was agreement up to 100% of cases between the macroscopic/microscopic observations and the MRI. This noninvasive technique gives rise to a follow-up of high reliability, which could be used to analyze the effects on grafted tumors of different forms of treatment. MRI scanning might spare animals in therapeutic experiments, because the tumor take is assessable before the therapeutic regimen is tested. However, access to instrumentation and the time involved in regularly scanning a lot of animals maybe a limiting factor for the use of this approach.

Orthotopic animal models of human GBM are often established by stereotaxic injection of cell suspensions (27, 34). Although the cell suspension methods of grafting can limit surgical trauma, it results in an overall take rate of less than 70%; it will take a long time to inject cell suspensions into animal brain with only 10µL suspensions pumped into the brain to cost at least 25 min, which is not suitable for mass production of anti-GBM experimental animal model. Furthermore, injected cells may flow back along the shaft of the needle and therefore into the arachnoidal space to disseminate. There is a large body of literature about GBM grafting as solid explants into brains of nude mice (18, 34), but the xenografts from human Grades III and IV astrocytomas were directly observed in mouse brain with an overall take rate of only 24%. Here, the patient GBM surgical tissues were taken into sc flanks of nude mice and passed from animals to animals before being xenotransplanted into the brain of nude mice, and secondary xenotransplantations of this sc mouse-adapted human tumor always resulted in 100% take rate. A striking advantage of our orthotopic xenotransplantation method is that the operation procedure often spends less than 3 min, providing massive animal model for further research in short time. We have previously demonstrated that our tumor development was rapid enough, often within 72 hr (data not shown), providing a reasonable time period for testing the most therapeutic modalities, while mean survival times as long as 1 year are observed when human GBMs are directly grafted into the animal brain. In addition, the volume of the sc tumors after a few weeks is several scores of intracerebral transplants, so a large number of mice can be grafted with tumor tissue from one original surgical specimen, making large homogeneous experimental lots of animals available for well-designed assays.

In summary, our report indicates that the intracranial xenografts derived from the orthotopic xenotransplantation of solid fragments of human GBM previously passaged within the mouse flank share biological characteristics with human GBM to a greater degree, particularly in relation to its growth characteristics, invasive properties, and *EGFR* gene overexpression. Considering their reproducibility, inexpen-siveness, and availability, the orthotopical xenografts constitute good preclinical models to test the novel antitumoral approaches involving chemotherapy and radiotherapy; they would also facilitate the identification of effective EGFR-targeted therapies, as well as the exploration of the new target aimed at the elements relevant to GBM invasion.
